# Flavonoid-Based Nanogels: A Comprehensive Overview

**DOI:** 10.3390/gels11040267

**Published:** 2025-04-04

**Authors:** Sergio Liga, Cristina Paul

**Affiliations:** Department of Applied Chemistry and Engineering of Organic and Natural Compounds, Faculty of Chemical Engineering, Biotechnologies and Environmental Protection, Politehnica University Timisoara, Vasile Pârvan No. 6, 300223 Timisoara, Romania; sergio.liga96@gmail.com

**Keywords:** nanogels, nanotechnology, flavonoids, nano-formulations

## Abstract

The growing field of nanotechnology has recently given much attention to nanogels, which are versatile formulas and have promising biomedical applications. Nanogels or nanohydrogels have undergone significant development in various fields of biomedical and industrial research to meet increasing demands, such as in pharmaceuticals, cosmetics, food, and genetic engineering. Nanogels that contain flavonoids, which are secondary metabolites found in plants, are starting to become distinctive and reveal their unique characteristics. The objective of the article is to provide a comprehensive overview of recent research articles on flavonoid-based nanogels, emphasizing the general aspects regarding nanogel formulation and structural characterization, as well as the advancements made in the biomedical field. In conclusion, this article outlines up-to-date developments in the synthesis, formulation, structural characterization, and therapeutic applications of flavonoid-based nanogels, emphasizing their important role in the field of nanotechnology.

## 1. Introduction

The development of new and more effective products is facilitated by nanotechnology in response to the current demand for enhancement of ongoing therapies and diagnostic examinations [1,2,3,4]. Drug development is being reshaped by the combination of nanotechnology and medicine, and nano-similar products are having a major impact on expanding treatment options, particularly in oncology [5,6,7]. The use of nanoparticles as delivery carriers has seen a significant increase and research in the past decade. But we also face challenges in nanomedicine development due to the complex biopharmaceutical behavior and pharmacokinetic and pharmacodynamic challenges of nanodrugs, which make it difficult to transition them from preclinical to clinical stages [8,9]. As a solution, nanogels are at the forefront of material science, connecting traditional polymers with cutting-edge nanotechnology. Due to ongoing advancements, these materials have revolutionized various fields, from precision medicine to sustainable technologies [3,10,11].

The term “nanogels” refers to hydrogels that are characterized by high levels of cross-linking, either in the form of monomers or polymers. The average size of nanogels typically ranges from 10 to 200 nanometers [12,13,14,15]. Their stability, biocompatibility, and high bioactive compound-loading capacity are due to their hydrophobic nature and physicochemical properties, which make them more favorable than other nanocarriers in the medical domain [13,15,16]. The unique architecture of these nanocarriers is characterized by a combination of interconnected structure and liquid phase, resulting in a dual, versatile character that possesses solid-like characteristics while also having liquid-like properties [15,16,17].

Nanogels are a highly versatile delivery vehicle that can enhance the efficiency of various compounds (e.g., natural compounds, peptides, drugs, oligonucleotides, nucleic acids, other compounds) [17,18,19,20,21,22]. The molecules released from nanogels can be managed by altering the amount or type of polymers, the charge of the bioactive compound or nanogel, or numerous stimuli–responsive parameters (e.g., pH, temperature, ions) [15,23,24]. The incorporation of nanoscale functionalities (e.g., nanoparticles, nanostructures, or bioactive compounds) into soft, three-dimensional polymeric networks is what makes nano-functional gels so effective in the medical field, tissue engineering, biosensing, and environmental remediation [14,21,23].

However, despite their promising pharmacological potential, flavonoids face several biopharmaceutical challenges that limit their transition to clinical applications. Flavonoids, a group of natural polyphenolic compounds that plants produce as secondary metabolites, with a benzo-γ-pyrone skeleton in their structure, generated through various synthesis pathways (e.g., phenylpropanoid pathway, shikimate pathway, and flavonoid pathway) possess numerous biological activities, making them an exciting discovery in the scientific community [25,26]. Based on the structure of flavonoids and depending on changes in their main structure, they can be classified into six major categories: (i) flavones, (ii) isoflavones, (iii) flavanones, (iv) flavonols, (v) flavanols, and (vi) anthocyanins [26,27,28]. Two major factors often limit their application in medical and industrial fields: (i) biopharmaceutical properties (e.g., poor solubility, low chemical stability and bioavailability, rapid metabolism) as mentioned before; and (ii) manufacture properties (e.g., low yield) [26,29].

According to their constituents, nanogels act as smart nanocarriers that have several biopharmaceutical advantages over other delivery carriers (e.g., micelles, solid-lipid nanoparticles, and polymeric nanoparticles): (1) improved biocompatibility and degradability; (2) increased capacity of drug/bioactive compound loading; (3) enhanced drug or bioactive compound solubility and stability; (4) reduced toxicity; (5) versatile design and functionalization; (6) particle nanoscale size and permeability; and (7) enhanced permeability and retention effect [30,31,32,33]. Flavonoid-based nanogels can be utilized to enhance the absorption and effectiveness of bioactive compounds, including hydrophobic drugs, by improving their solubility and bioavailability. Another fact is that flavonoids’ bioactivity can be synergized with encapsulated drugs, enhancing therapeutic efficiency in diseases (e.g., cancer, neurodegenerative disorders, cardiovascular conditions) [34,35,36]. Flavonoid-loaded nanogels offer a potentially more cost-effective and sustainable alternative to synthetic polymers used as nanocarriers [37]. However, nanogels also have certain disadvantages, including high costs, manufacturing challenges (for large-scale production), and limited clinical therapies. Moreover, the use of model-informed drug development techniques that are based on models (e.g., Quality-by-Design, in vitro–in vivo correlations (IVIVC), and the physiologically based biopharmaceutics model) can prevent these disadvantages [5].

Unfortunately, current studies on flavonoid-loaded nanogels are still ongoing. Most of the literature-based studies focus on the investigation of only a few flavonoids encapsulated in nanogels (e.g., quercetin, naringenin, apigenin). Our proposal was to conduct a review that would incorporate the general principles of nanogel composition and formulation, as well as the most recent studies carried out in this innovative field, as a solution to encapsulation in nanogels, and to improve the biopharmaceutical properties of flavonoids.

## 2. Methods

**Figure 1:**
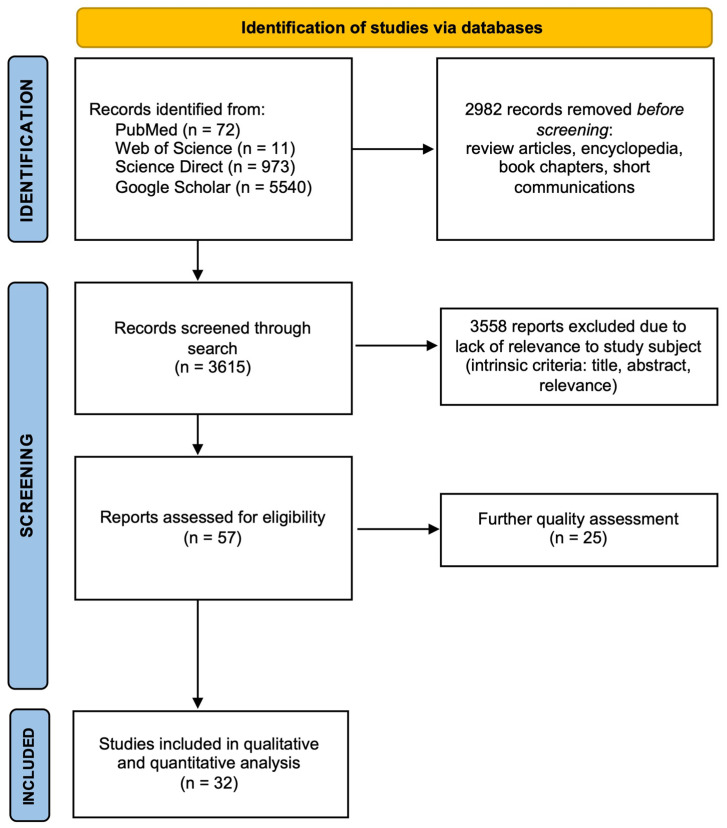
PRISMA flow-diagram of the bibliographic research.

This study is a comprehensive review of the literature that includes information on flavonoid-based nanogels. To gather the data, the scientific literature was searched in various databases, including PubMed, Web of Science, Google Scholar, and Science Direct. The criteria for selecting the material were as follows: a search was conducted on the PubMed database [38], using the keywords “nanogels, flavonoids” OR “flavonoid-based nanogels”. The results returned 72 papers up to 2025. A second search was conducted on the Web of Science, and the results returned 11 scientific papers. A third search was conducted on the Science Direct database [39], using the same keywords. This returned 973 articles. A fourth search was conducted on the Google Scholar database [40], using the same keywords; this returned over 5540 articles; these were summarized and critically discussed to provide a consistent overview (Figure 1). This meta-analysis was performed according to the PRISMA guidelines 2020 [41].

**Figure 2:**
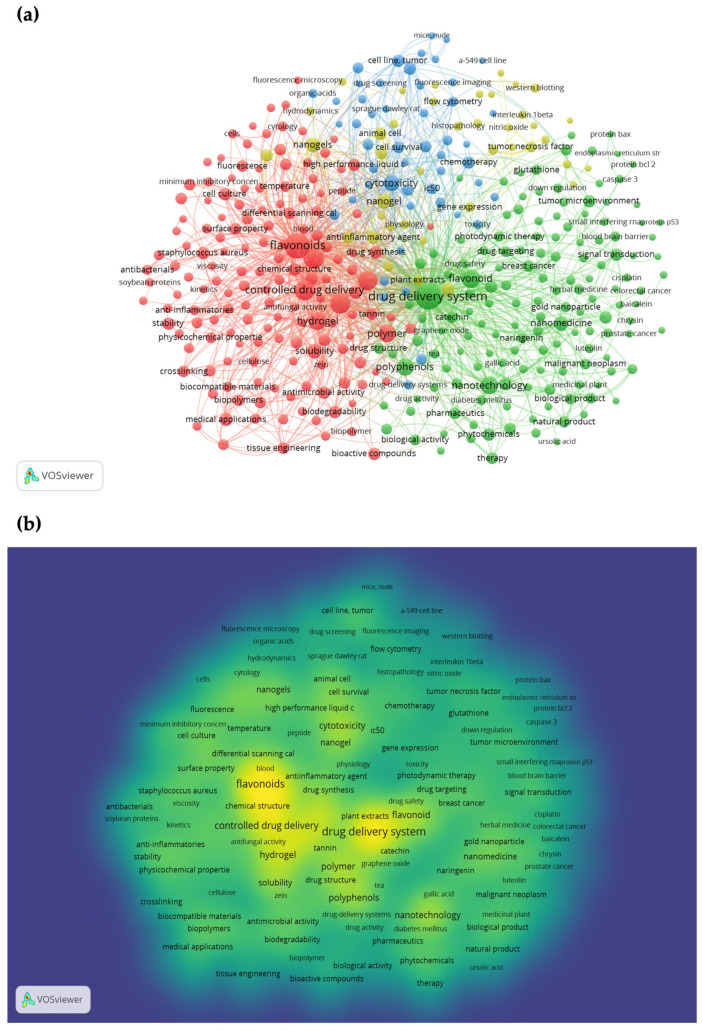
Keywords co-occurrence analysis: (**a**) VOSviewer network visualization map of keyword co-occurrence, and (**b**) VOSviewer density visualization map.

The colored cloud depicted in Figure 2 was obtained by VOSviewer software (version 1.6.20, www.vosviewer.com, accessed on 18 March 2025) using the Scopus online database. During the bibliographic search (*n* = 1374 documents), the same main keywords “nanogels, flavonoids” were used. We conducted co-occurrence (map) analysis based on the text data from the title, abstract, and keyword of the documents found. Figure 2a illustrates the co-occurrence network between keywords (indicated by the curved lines), which refers to the frequency (indicated by the size of the circles) of their occurrence in a document. In our analysis, the minimum occurrence of a keyword was defined as five, and four clusters were obtained. Flavonoids, drug delivery system, controlled drug delivery, nanotechnology, hydrogel, and nanogels were the main keywords included in the colored cloud, which align with the main objectives of our review. According to density visualization map results, the color of a keyword can be used to indicate its occurrence, with lighter colors indicating increased research activity and faded colors indicating decreased activity (Figure 2b). The keyword ‘nanogels’ appears as faded and almost blends into the green background, indicating that there has been a limited amount of research conducted on this keyword related to flavonoids.

## 3. General Classification and Formulation Techniques for Nanogels

### 3.1. Classification of Nanogels

#### 3.1.1. Based on Matrix Composition

The composition of functional nanogels is a decisive factor in the formulation, pharmacokinetic behavior, and application of drug/bioactive molecule-loaded gels in therapy. The basic constituents of the nanogel matrix include natural polymers (e.g., collagen, gelatin, hyaluronic acid (HA), chitosan, alginate, agarose, carrageenan), synthetic polymers (e.g., PEG and polyglycolic derivatives, polyvinyl alcohol (PVA), polyacrylamide, poly(lactic acid) (PLA), poly(ε-caprolactone), poly(lactic-co-glycolic acid) (PLGA)), or hybrid polymers (e.g., chitosan-PEG, alginate-PEG) [20,32,42,43,44].

**Figure 3:**
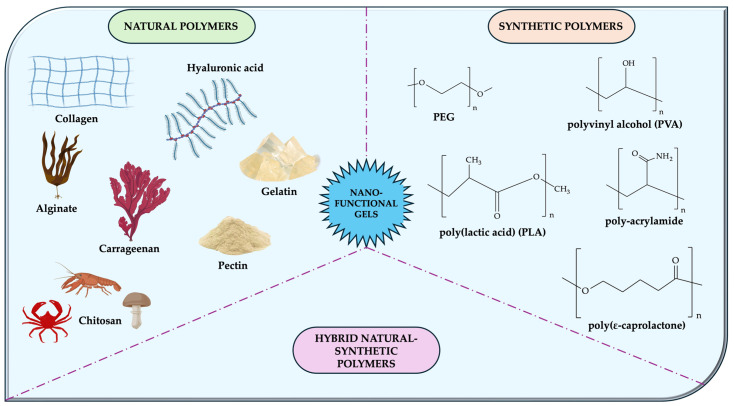
The main components found in functional flavonoid-loaded nanogels. This image was created using BioRender (BioRender.com, accessed on 7 January 2025).

The most common components in nano-functional gels loaded with flavonoids are shown in the figure below (Figure 3).

The classification of nanogels is determined by the type of polymeric molecules utilized in their formulation (Table 1), a factor which exerts a significant influence on their properties, drug delivery capabilities, and applications. The composition classifications are divided into natural polymer-based, synthetic polymer-based, and hybrid functional nanogels [20,43].

#### 3.1.2. Based on Formulation Technique

**Figure 4:**
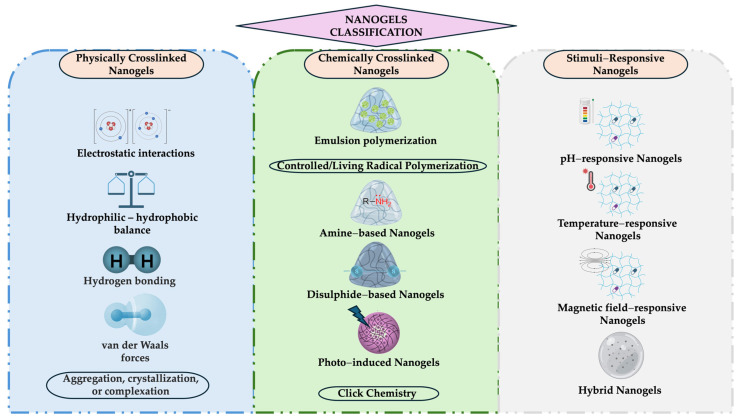
Classification of nanogels according to the formulation technique. This image was created using BioRender (BioRender.com, accessed on 7 January 2025).

The nanogel classification, according to technique formulation, can be divided into physical, chemical, and stimuli-responsive categories, as shown in Figure 4.

##### Physically Crosslinked Nanogels

This category is also referred to as pseudo-gels, wherein cross-linking occurs through a physical process and is influenced by the polymer’s characteristics, including composition, temperature, concentration, and medium ionic strength [13,14,20]. The physical processes involved, as depicted in Figure 4, encompass electrostatic interactions, hydrophilic–hydrophobic balance, hydrogen bond, van der Waals forces, and solid-state effects (e.g., aggregation, crystallization, complexation, etc.) [20,73,74]. The physically crosslinked process is driven by multiple weak non-covalent interactions, including hydrogen bonding, electrostatic interactions, van der Waals, and hydrophobic interactions [74]. The synthesis of functional nanogels through physical crosslinking offers distinct advantages over chemical strategies, exhibiting mild synthesis conditions and the potential for adjusting their physicochemical properties through the regulation of polymer concentration and experimental conditions [22,75].

##### Chemically Crosslinked Nanogels

**Figure 5:**
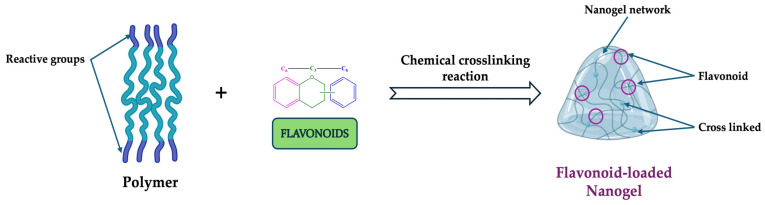
Generic chemically crosslinking reaction (emulsion polymerization, radical polymerization, click chemistry, and photo-induced reaction) protocol for flavonoid-loaded nanogels. This image was created using BioRender (BioRender.com, accessed on 7 January 2025).

The purpose of chemically cross-linking nanogels (Figure 5) is to provide stability and functional properties by using covalent bonding between polymer chains [8]. This type of nanogel has a variety of reactions that offer advantages in terms of drug/bioactive molecule specificity, efficiency, and stability. The strength of these linkages is found to be significantly influenced by the functional groups present in the nanogel molecules and the specific cross-link locations [76,77,78].

##### Stimuli-Responsive Nanogels

Recent years have seen a surge in the popularity of personalized therapy, which in turn has led to the development of stimuli-responsive nanogels and smart biomaterials. In contrast to conventional gels, smart or stimuli-responsive nanogels respond to external stimuli (e.g., pH, temperature, magnetic field, light) and offer more efficient and valuable properties to the medical and industrial fields [24,79,80,81,82,83]. These stimuli-responsive nanogels can respond to triggers with reversible and reproducible macroscopical changes and can return to their original initial state after the triggers are removed [14,80,84].

Deng et al. prepared thermosensitive nanogels by copolymerizing N-isopropylacrylamide (NIPAM) with N-hydroxymethyl acrylamide (PMAM) or 2-hydroxyethyl methacrylate (HEMA) monomers in response to different concentrations of (−)-Epigallocatechin-3-gallate and ethyl gallate, at temperatures ranging from 5 to 45 °C. Additionally, the evidence shows that polyphenols and the hydrogel network. with diverse compositions, have distinct molecular interactions [85].

### 3.2. Formulation Techniques for Nanogels

Advanced drug delivery systems known as nano-functional gels combine the advantages of nanotechnology with gel matrices. The objective of incorporating flavonoids into these systems is to improve their solubility, chemical stability, bioavailability, and controlled release [16]. The following are different strategies for creating nano-functional gels that are loaded with flavonoids.

#### 3.2.1. Emulsion Polymerization Technique

In the case of the emulsion polymerization technique, an emulsifying monomer (which is usually not water-soluble) is mixed with an aqueous solution of a surfactant and then anchored by surfactants at the monomer–water interface (equal to or above their critical micelle concentration) after different mechanical stimuli are applied [22,86,87,88,89]. The initiation of the emulsion polymerization technique requires persulfates or cationic initiating species (e.g., 2,2′-azobis(2-methylpropionamidine) dihydrochloride) [90].

Over time, the literature has classified this technique into the mini-emulsion polymerization technique, microemulsion polymerization technique, and reverse (W/O) microemulsion polymerization technique [22,86,89].

The microemulsion technique is a semi-heterogeneous system that consists of two insoluble aqueous and oil phases and allows monomer droplets to transform directly into polymer particles (particle size range between 50 and 500 nm), compared to emulsion polymerization (generally above 100 nm) [90].

In the case of the reverse (W/O) microemulsion polymerization technique, which is an effective method for producing nanogel-controlled polymers with a high molecular weight [90,91], nanogels can be produced with low viscosity and a high content of bioactive molecules. On the other hand, in the mini-emulsion polymerization technique, monomer droplets are directly transformed into polymer particles via oil-soluble initiators (droplet nucleation), using ultrasonic waves or a high-speed homogenizer [86,92].

#### 3.2.2. Solvent Evaporation Technique

**Figure 6:**
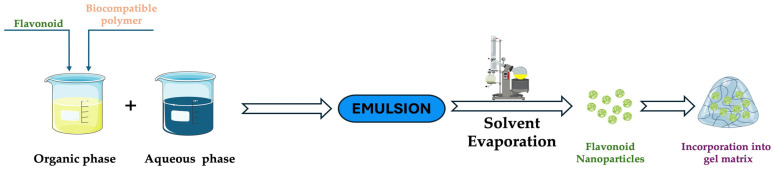
Schematic protocol for the solvent evaporation technique. This image was created using BioRender (BioRender.com, accessed on 7 January 2025).

The solvent evaporation technique involves removing both the flavonoid and a biocompatible polymer from an organic solvent. Under reduced pressure (using a rotary evaporator) or at room temperature, the solvent is evaporated to form nanoparticles that encapsulate the flavonoid (Figure 6). After this process, the nanoparticles can be incorporated into a gel matrix for controlled release, such as hydrogels, stimuli-responsive gels, or ionic crosslinking gels [19,21].

#### 3.2.3. Ionic Gelation Technique

**Figure 7:**
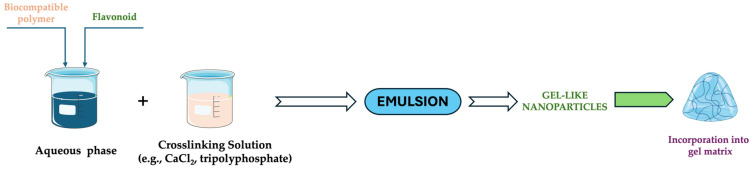
Schematic protocol for the ionic gelation technique. This image was created using BioRender (BioRender.com, accessed on 7 January 2025).

The ionic gelation method is widely used to prepare nanogels, particularly using biocompatible and biodegradable polymers (e.g., alginate, chitosan, and pectin). The principle of ionotropic gelation is used to create gel-like nanoparticles by crosslinking the polymer chains in the presence of divalent ions (e.g., Ca^2+^) [19,21,93]. In our case, the ionic gelation method involves dissolving the polymer in an aqueous solution, followed by adding the flavonoid. After the flavonoid and polymer solution are prepared, a divalent ion solution can be added to crosslink the polymer. Controlled release and enhanced bioavailability are achieved through the formation of nanoparticles that encapsulate the flavonoid inside the polymer matrix (Figure 7).

Szulc-Musioł et al. mixed sodium alginate with water, glycerol, and a 0.5% solution of calcium chloride (crosslinking agent) at ambient temperature, after adding quercetin, to obtain a semi-solid, homogeneous nanogel. It was observed that the nanogel forms a typical cone on the fingertip with a higher hardness (0.0520 ± 0.001) and a pH 5.6 [94].

Salah et al. developed a chitosan gel functionalized with cinnamaldehyde oil and flavonoid extract. The orange peel samples were processed by using ethanol 100% (*v*/*v*) at 37 °C and stirred magnetically for six hours, at 200 rpm. The purification process involved filtering the extracts through Whatman filter paper to remove peel particles and then subjecting it to an AB-8 macro-porous resin column to separate the purified extract with 80% ethanol. Following, various levels of flavonoid extract were added to nanogel using the ionic gelation technique, with phytic acid acting as a cross-linker agent. The results demonstrated high encapsulation efficiency for both components and exhibited significant antimicrobial inhibition of microbial growth for *Penicillium expansum*, *Staphylococcus aureus*, *Escherichia coli*, and *Bacillus cereus* [95].

Ding et al. demonstrated that zein–alginate nanogels loaded with curcumin can be preserved and processed more effectively as powder formulations, which eliminates the issue of high transportation expenses. Additionally, the nanogels exhibit superior photostability and thermal stability, more efficient encapsulation, and show major advantages in controlling the release of curcumin [96].

Kaushal et al. investigated the subsequent release of alginate hydrogels via in vitro gastrointestinal environments and their ability to support flavonoid-laden poly-lactic-co-glycolic acid (PLGA) nanoparticles. Calcium chloride was used as a cross-linking agent for the ionic gelation of sodium alginate suspension that contained flavonoid–PLGA nanoparticles. According to the study’s findings, flavonoids were released during the intestinal phase, as indicated by mass spectrometry, in vitro release studies, and microstructure analysis [97].

#### 3.2.4. Photopolymerization Technique

The photopolymerization technique relies on UV or visible light to break apart a photo-initiator (e.g., phosphine-oxides, phosphinates, biogenic sources) into radicals, causing the bonding of monomers into a crosslinked network, leading to the formation of nanogels [98,99,100,101]. Adjusting the light intensity, exposure time, or photo-initiator concentration is necessary for achieving efficiency and good mechanical properties.

For example, Li et al. developed nanogels loaded with chrysin through a photopolymerization technique, using a green LED as an emission source at 540 nm. According to the findings, chrysin inhibits monomer polymerization, leading to a decrease in double bond conversion in the system [102].

#### 3.2.5. Microfluidic Technique

**Figure 8:**
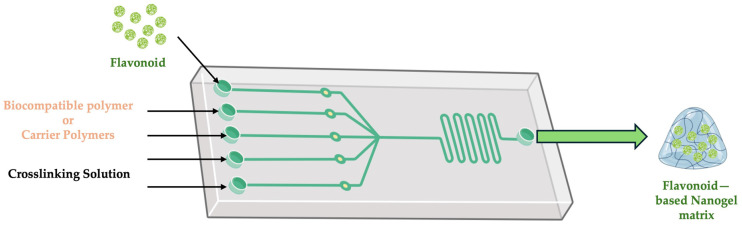
Schematic protocol for the microfluidic technique. This image was created using BioRender (BioRender.com, accessed on 18 March 2025).

In microfluidic systems, fluids are manipulated at the microscale in microchannels, which allows for rapid mixing, precise control over reaction conditions, and continuous production of nanogels [103,104,105]. Microfluidic technology (Figure 8), unlike batch processes, can produce continuously, making it suitable for large-scale production [106]. Continuous monitoring, optimization, and control of the nanogel formulation process can be achieved by integrating microfluidic systems with automated systems. Such systems can be designed to modify variables according to real-time sensor feedback (e.g., flow rates, concentration, pH) to ensure optimal synthesis conditions throughout production [104,107].

## 4. Structural Characterization of Flavonoid-Loaded Nanogels

To obtain detailed information, flavonoid-loaded nanogels are characterized using existing physico-chemical methods and model-informed tools that have been used in nanotechnology. Table 2 consists of a list of techniques and some important insights that are frequently employed.

The physical and chemical characterization of flavonoid-loaded nanogels is challenging because of flavonoid’s structural complexity, diverse glycosylation, methylation or polymerization forms, and the possibility of degradation (oxidation, hydrolysis, UV light exposure). Analytical methods often encounter difficulties, like co-elution, low ionization efficiency, and matrix interferences, making it difficult to accurately identify and quantify. To overcome these challenges, hyphenated techniques, such as Liquid Chromatography–Mass Spectrometry (LC–MS), High-Resolution Mass Spectrometry (HRMS), and Nuclear Magnetic Resonance (NMR) spectroscopy are widely employed [114]. The use of these techniques enhances structural elucidation, enhances detection sensitivity, and facilitates high-throughput screening. The application of these techniques to flavonoid-loaded nanogels helps ensure precise encapsulation efficiency, stability assessment, and controlled release profiling, which makes it easier to develop for the biomedical, diagnostics, pharmaceutical, and industrial fields.

## 5. Applications of Flavonoid-Loaded Nanogels in Therapy

### 5.1. Delivery Systems in Cancer Therapy

Flavonoid-based nanogels have a significant use as smart modalities to cross biological barriers, particularly in the field of cancer therapy [124,125,126]. The nanogel matrix also acts as a solution by encapsulating flavonoids in their hydrophilic and hydrophobic domains, which ensures a more controlled and sustained release [127,128]. Flavonoid concentration fluctuations are minimized, dosing frequencies are reduced, and therapeutic efficacy is enhanced through controlled release. In addition, the nanogel matrix ensures the stability and activity of flavonoids in biological systems by protecting them from enzymatic degradation [89]. These active biomolecules are ideal candidates because they have therapeutic activities that make them ideal for cancer treatment, as chemotherapy drugs cause adverse reactions of varying severity and can also kill normal cells in the human body.

Mangalathillam et al. developed transdermal nanogels using biocompatible and biodegradable chitin with curcumin. According to their findings, the formulated nanogels have specific benefits for the treatment of melanoma, the most common and severe form of skin cancer. Cytotoxic experiments were conducted with the use of MTT assay on human dermal fibroblast cells and human melanoma cell lines (A375). Additionally, it was discovered that curcumin conjugation decreases the quantity of free reactive functional groups present on the chitin nanogels [129].

Choi et al. prepared hyaluronic acid-based nanogels conjugated with dihydroxyflavone to evaluate cellular uptake and antitumoral efficiency. The formulation includes a methanolic solution of dihydroxyflavone, which was added to an aqueous hyaluronic acid solution with different concentrations of 4-(4,6-dimethoxy-1,3,5-triazin-2-yl)-4-methylmorpholinium chloride (a conjugation agent for esterification and crosslinking). According to reports, the nanogels are approximately 150 nm in size, which is within the ideal range for efficient cellular uptake, particularly for passive targeting through the enhanced permeability and retention effect in tumors. After applying these nanogels to NIH/3T3, HepG2, and HeLa cell lines, it was discovered that the nanogels had better cellular uptake and antitumoral effects among the samples. Based on the release profile, which showed a sustained release over 48 h and a slow and controlled release of dihydroxyflavone, it can also be concluded that these nanogels can have a long-term therapeutic effect [130].

The study by El-Kholy et al. provides valuable insights into the synthesis and characterization of quercetin-loaded carboxymethylcellulose nanogels. The particle sizes ranged from 93 nm to 591 nm, and the zeta potential values ranged from −41 mV to −25 mV. According to the study, the nanogel’s swelling ratio reached 53% within 24 h, and then increased to 61.46% and 63.45% when quercetin was added at 25 mg and 50 mg. Adding 75 mg of quercetin led to a reduction in the swelling ratio to 60.11%. The nanogels exhibited sustained pH-responsive drug release for up to 7 h. Cytotoxic tests revealed that the formulations had a cytotoxicity score of 56.6% against MCF-7 (breast cancer cells) and 70% against HepG2 (liver cancer cells). In vitro release results further demonstrate the tunable release behavior of these nanogels in different environments, which can be optimized for specific therapeutic needs [131].

For example, pH-sensitive nanogels are designed to release drugs selectively in acidic environments (e.g., the tumor microenvironment) [132]. El-Adl et al. explored the development and evaluation of a pH-sensitive pectin-based nanogel designed for targeted and controlled rutin release in cancer therapy. The nanogel exhibited a pH-sensitive behavior, releasing flavonoid rutin selectively in response to environmental pH changes. The strong hydrogen bonding of the nanogel structure results in minimal rutin release at acidic pH levels (from pH 1 to 3). At pH 4, the decomplexation of pectin and polyacrylic acid resulted in swelling of the nanogel and an increase in rutin release. Under alkaline conditions (pH 5 to 8), the presence of sodium counterions induces a charge screening effect, reducing nanogel porosity and subsequently decreasing rutin release by approximately 66%. The authors emphasize the pectin–polyacrylic acid nanogel as a reliable method to deliver flavonoid rutin, enabling controlled release, increased bioavailability, and enhanced anticancer activity, resulting in a promising candidate for targeted cancer therapy [133].

### 5.2. Therapeutic Systems

Jiang et al. proposed the use of two inhibitors (curcumin and epigallocatechin-3-gallate) to modify hyaluronic acid and to study their synergistic effects, which are known for their ability to inhibit the amyloid β-protein from Alzheimer’s disease. According to the measurements, the dual inhibitor-modified hyaluronic acid nanogels were about 150–250 nm in size and exhibited a zeta potential of +30 mV. Neuroprotection was achieved by the dual nanogels’ ability to reduce oxidative stress in SH-SY5Y cells induced by Aβ treatment. Based on the results, the creation of dual inhibitor nanogel is a promising strategy for the creation of potent agents against amyloid β-protein aggregation and cytotoxicity [134].

Samadian et al. aimed to assess the effects produced on the K562 cell line of chronic myeloid leukemia by apigenin and the nanogel created by combining it with stearate-chitosan. The results indicated that the viability of K562 cells in the presence of free-apigenin and apigenin-loaded nanogel varied depending on the dose and exposure time. The nanogel formulation measured a size between 120 and 150 nm, with a zeta potential of around +30 mV, indicating good stability for drug delivery. However, cells treated with an apigenin-loaded nanogel experienced a greater degree of apoptosis (at 72 h is 66.37%) than those treated with free-apigenin (60.21%) [135].

Chen et al. investigated for the treatment of acute lung injury, a novel alginate–quercetin based “material-drug” structural inhalable nanogel. They produced a water-soluble nanogel system that stabilizes quercetin through hydrogen bonding through an emulsion polymerization method. The quercetin loading and encapsulation efficiency of the nanogel were 0.92 ± 0.02%, respectively 97.7 ± 1.2%. The nanogel was examined for cell viability, uptake, and protective effects on the human pulmonary carcinoma cell line and the murine macrophage RAW264.7. The inhalable quercetin nanogel has demonstrated a reduction in the mRNA and protein expression of inflammatory cytokines, leading to a reduction in pulmonary inflammation, a promising therapeutic approach for acute lung injury [136].

Min-Rui Tai et al. investigated the development of biomimetic triumvirate nanogel complexes via the self-assembly peptide and flavonoid hesperidin. In vitro studies on hesperidin loading and release showed that it has a high encapsulation efficiency (>85%) and a pH-responsive release system, with faster release observed under acidic conditions (pH 5.5) that simulate tumor microenvironments. The biocompatibility of the nanogel was confirmed through cytotoxicity assays (MTT) on human cells, with cell viability above 90% even at high concentrations. Based on these findings, it seems that biomimetic nanogels based on hesperidin have the potential for targeted drug delivery and tissue engineering [137].

In another study conducted by Deghiedy et al., their research resulted in the synthesis of nanogels with Fisetin-loaded with pluronic-2-Acrylamido-2-methylpropane sulfonic acid, by using gamma radiation. All nanogel formulations exhibited negative ζ-potential values within the range of −15 to −43 mV, PDI between 0.07 and 0.771, mean particle sizes between 130 and 1000 nm. The nanogel modulates oxidative stress, inflammatory markers alternation, cellular damage, and apoptotic markers alternations induced by AlCl3/D-galactose. The histopathological findings also revealed the occurrence of neurofibrillary tangles. The neurocognitive impairments associated with Alzheimer’s disease were effectively mitigated by using fisetin-loaded nanogel [138].

Naeem et al. wanted to develop a nanogel membrane comprised of sodium alginate, polyvinyl alcohol, acrylic acid, and gallic acid for the treatment of skin wounds. The free radical polymerization technique was adopted for the preparation of the nanogel membrane. Physicochemical tests (FT–IR, TG, DSC, and electron microscopy analysis), biodegradability tests, and mechanical measurements were performed on this nanogel membrane. It was found that the nanogel had excellent antioxidant potential and strong antimicrobial properties towards both gram-positive (*E. coli* and *S. aureus*), and gram-negative (*P. aeruginosa*) microbial strains. However, to clarify the mechanism of wound healing effects, additional in vitro and in vivo studies are necessary [139]. Table 3 presents relevant studies regarding flavonoid-loaded nanogels.

## 6. Challenges and Future Directions

Although flavonoid-loaded nanogels have been shown to be promising in preclinical studies, they have not yet been approved for clinical use. Reproducible and scalable manufacturing difficulties, as well as maintaining desirable biopharmaceutical properties, are among the key challenges that hinder clinical translation.

Firstly, flavonoids’ limiting biopharmaceutical factors (e.g., bioavailability and stability, poor absorption, and rapid metabolism in the human body) possess a major obstacle that can be overcome by developing new nanogel systems. Despite sharing the same challenges, all flavonoid groups have distinct biopharmaceutical limitations:

▪ Flavones and flavonols have poor water solubility, are rapidly metabolized in the liver, and circulate as methyl, glucuronide, and sulfate metabolites [147,148].
▪ Flavanones have poor permeability, are resistant to degradation in the stomach and small intestine and are deconjugated upon reaching the proximal colon [149,150].
▪ Anthocyanins have low bioavailability, are generally degraded at higher pH levels, and are unstable at neutral pH levels [151,152].
▪ Isoflavones have low bioavailability, making it difficult for them to pass through the intestinal epithelium and be absorbed, leading to weak biological activity [153].

**Figure 9:**
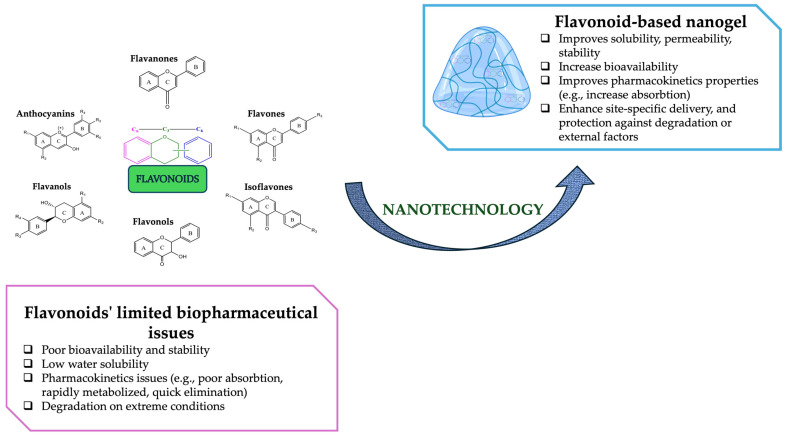
Nanotechnology approaches to improving biopharmaceutical activity and enhancing the applicability of flavonoids. This image was created using BioRender (BioRender.com, accessed on 28 March 2025).

The incorporation of flavonoids into nanosized delivery systems improves their physicochemical and biopharmaceutical properties, particularly their bioavailability and solubility profile, resulting in enhanced therapeutic potential, site-specific delivery, and protection against degradation (Figure 9) [148,154,155].

In this regard, nanogels offer a highly promising formulation strategy, facilitating improved solubility, permeability, stability, and controlled pharmacokinetic behavior. Their targeted delivery is facilitated by their precise nanoscale structure, which also allows for a reversible solgel transition to ensure sustained interaction with diseased tissues, leading to the maximization of therapeutic benefits and the increase of their apparent solubility through their dissolution rate and bioavailability [155,156]. This characteristic cross-linked structural network of nanogels allows the flavonoid to remain entrapped inside such a three-dimensional matrix for controlled and site-specific release. 

Furthermore, changing the size of the pores in the nanogel network can enable flavonoid molecules to interact with the polymer chain and ensure accurate encapsulation [154,157]. The permeability of nanogels is thus enhanced by their ability to aid in muco-adhesion, guarantee long-term retention at absorption sites, and promote paracellular transport by opening tight junctions [158,159,160].

Following this, comprehensive toxicology studies will need to be carried out and biocompatible nanogel systems to be developed. It is imperative that future research investigates the long-term stability of these systems, with particular emphasis on the degradation of flavonoids and their interactions with biological components, such as human cells. On the other hand, integrating flavonoid-based nanogels with model-informed drug development tools and advanced imaging techniques for real-time monitoring of drug release and therapeutic progress could be a future development for these nanogels. Smart nanogels, dual-drug delivery systems, or multi-functional nanogels that incorporate a range of bioactive substances may be future directions to improve therapeutic efficacy, reduce resistance, and improve patient outcomes, particularly in complex diseases. The increasing computational power available to researchers will facilitate more in-depth investigations of these complex systems, which will ultimately lead to their clinical application.

## Figures and Tables

**Table 1:** Characteristics of polymeric molecules utilized in the nanogel formulation.

Basic Constituent	Type of Polymer	Characteristics	References
Natural polymers	Collagen	ensure biocompatibility with human tissues by mimicking the extracellular matrix ensure biodegradability promote cell adhesion, migration, proliferation, essential for skin and cartilage repair crosslinking enhancements	[45,46,47,48,49]
	Alginate	good mechanical strength, and degradation rate (because of its β-d-mannuronic acid and α-l-guluronic acid monomers) good biopharmaceutical properties (e.g., stable at acidic pH, maintain an optimal skin moist environment) fast gelling rate	[49,50,51,52,53]
	Hyaluronic acid	good biodegradability, high-water retention rate, and hygroscopicity targeted delivery capabilities and efficient carriers for drugs, natural compounds, peptides, proteins, and nucleic acids high biocompatibility (form a hydrogel matrix that keeps wounds hydrated, accelerating healing, and promoting fibroblast and keratinocyte proliferation)	[54,55,56]
	Chitosan	good biocompatibility, biodegradability large surface area, and adhesiveness stimuli responsiveness high molecule loading capacity nontoxicity	[57,58,59,60]
	Gelatin	unique physico-chemical properties (large capacity for surface loading, emulsifier, thermo-reversible gel-forming, transparency, cold water insolubility, and melting point at body temperature)	[61,62,63]
Synthetic polymers	PEG, polyglycolic derivatives	safe and non-toxic polymer steric stabilization assure high biocompatibility and hydrophilicity can be blended with natural polymers (e.g., chitosan, alginate, hyaluronic acid) to modify its physical and chemical cross-linking properties	[50,64,65,66]
	polyvinyl alcohol (PVA)	biocompatible water-soluble stabilizer steric repulsion (stabilize polymer particles)	[67,68]
	polyacrylamide	good hydrophilicity non-toxicity high water retention capacity assure good encapsulation rate of hydrophilic and hydrophobic bioactive molecules	[69,70]
	poly(lactic-co-glycolic acid) (PLGA)	good biocompatibility rate good biodegradability rate	[71,72]

**Table 2:** Characterization techniques for flavonoid-based nanogels.

Flavonoid-Based Nanogel Characterization	Key Characteristics	References
Swelling Measurement	fundamental intrinsic property large surface area high fluid exchange capacity with the environment swelling rate depends on endogenous (e.g., enzymes, pH, etc.) or exogenous stimuli (e.g., temperature, light, magnetic fields) the main characteristic parameter for swelling is molecular mass between crosslinks (Mc) *Swelling Capacity (%)* = W_s_ − W_d_/W_d_, where W_s_ (ratio of the weight of swollen nanogel) and W_d_ (weight of dry nanogel)	[24,31,57,108,109]
Mechanical Measurements	nanogels possess the mechanical qualities of both solid and liquid substances, and have a significant impact on both in-vivo and in-vitro behaviors the main measurement method is dynamic mechanical analysis (DMA)	[13,110]
Electron Microscopy Measurements	Nanogels can be studied for determining morphological characteristics using a variety of robust and well-established techniques: scanning electron microscopy (SEM) transmission electron microscopy (TEM) atomic force microscopy (AFM) laser scanning confocal microscopy (LSCM) micro-computed tomography scanning tunneling microscopy (STM)	[14,75]
X-ray Scattering Techniques	X-ray scattering (SAXS) wide-angle X-ray scattering (WAXS)	[14,75,111]
Rheology	Any route of administration has significant effects on the therapeutic efficacy and biopharmaceutical performance of incorporated flavonoids due to the change in viscosity of nanogel	[75]
Thermal analysis	provide a comprehensive understanding of the thermal characteristics of a nanogel nanogel’s biocompatibility could be impacted by thermal transitions (e.g., glass transition temperature, initial decomposition temperature, degree of crystallinity, etc.) main popular techniques for nanogels are thermogravimetric analysis (TGA) and differential scanning calorimetry (DSC)	[13,44]
Dynamic Light Scattering (DLS)	measures the hydrodynamic diameter, and determines size distribution and polydispersity index (PDI) by calculating the diffusion coefficient of particles in a liquid medium evaluates aggregation tendencies under different storage or biological conditions monitors size changes due to drug loading within nanogels	[112,113]
Hyphenated Techniques (LC–MS, HRMS, NMR)	Liquid Chromatography-Mass Spectrometry (LC-MS) is an effective instrument for analyzing nanogels’ size, composition, and molecular weight distribution, as well as quantifying the encapsulation efficiency of flavonoids loaded within the nanogel matrix High-Resolution Mass Spectrometry (HRMS) offers measurements with high accuracy to verify the structure of flavonoids, polymers, or other molecules present in the nanogel, as well as to detect and quantify small molecules that may be incorporated into the nanogel formula, such as stabilizers, surfactants, or additives NMR spectroscopy offers comprehensive knowledge about the molecular structure of flavonoids, establishes their identity and purity, and can also to assess flavonoid-loaded nanogel stability over time	[114,115,116,117]
Model-Informed Drug Development (MIDD)	*In Vitro–In Vivo Correlation (IVIVC) model*: ○ predictive mathematical model describing the relationship between the in vitro properties of an oral dosage form and the relevant in vivo response ○ optimized formulation parameters, predicted bioavailability, and reduced the need for extensive clinical trials. ○ the model can establish an in vitro drug release profile (e.g., dissolution and release mechanism) for flavonoid-based nanogels, calculate in vivo pharmacokinetic parameters, and optimize nanogel formulations to enhance therapeutic effectiveness and controlled flavonoid delivery *Quality-by-Design*: ○ a modern tool aimed at systematic drug development by employing statistical, analytical, and risk management methodologies in the design, development, and manufacturing of drug products ○ could tackle manufacturing and flavonoid loading challenges (e.g., flavonoid release rate from the nanogel-based system, mechanical strength), and desired quality attributes of the final flavonoid-based nanogel formulation	[117,118,119,120,121,122,123]

**Table 3:** Relevant studies regarding flavonoid-loaded nanogels.

Flavonoid	Results	References
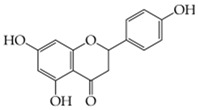 **Naringenin** (5,7-dihydroxy-2-(4-hydroxyphenyl)-chroman-4-one)	Nanogel polymer: Chitosan Nano-emulsion technique Drug globule size = 15.69 ± 0.737 nm, PDI = 0.330, zeta potential = 8.33 ± 3.09 mV, pH = 5.84 ± 0.060, viscosity 54.66 ± 1.52 cP. The presence of chitosan, a cationic polymer with a positive charge on its surface, enhances the interaction between negatively charged stratum corneum and improves the parameters of adhesion strength and force. The formulation’s positive effects on wound healing can be attributed to its low cytotoxicity in fibroblast cells and the accelerated wound healing potential.	[140]
	Nanogel polymer: Polyvinylpyrrolidone (PVP) Nanoprecipitation technique XRD analysis shows that the crystal structure of naringenin became amorphous during formulation. Nanogel exhibits significant antioxidant and anti-inflammatory properties without affecting any biochemical or hematological parameters. The inflammatory and stress signaling pathway genes were found to be unchanged at all tested doses compared to the control group, as evidenced by histopathological studies.	[141]
	Nanogel polymer: Chitosan and chondroitin sulphate Polyelectrolytic complexation and co-precipitation techniques pH = 5.5, PDI = 0.273 ± 0.008, zeta potential = + 19.36 ± 0.27 mV, EE% = 81.80 ± 1.23%, LC% = 27.20 ± 0.89%. High transparency of the formulation (N3@NAR/β-CD) indicates its suitability for intravitreal injections without causing visual disturbances, and reduced oxidative stress. The formulation was confirmed to be biocompatible by in vitro cytotoxicity assay on HUVEC cells.	[142]
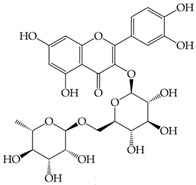 **Rutin** (3,3′,4′,5,7-pentahydroxyflavone-3-rhamnoside)	Nanogel polymer: poly(ethylene glycol)-diglycidyl-ether (PEGGE) Microemulsion technique Hydrolytic size distribution = 548 ± 8.9 nm, PDI = 0.452 ± 0.041, zeta potential between −3.1 and −32.35 mV. The alpha-glucosidase enzyme activity gradually increased between 50 and 750 µg/mL in the presence of rutin-loaded nanogels, with the fractional activity changing from 1.2 ± 0.3 to 1.5 ± 0.7. Rutin-loaded micro/nanogels showed greater blood compatibility than free-rutin, even at high concentrations, with no significant effects on the biological functions of fibrinogen due to molecular interactions. These findings have shown that micro/nanogels loaded with rutin are more appropriate for intravenous applications.	[143]
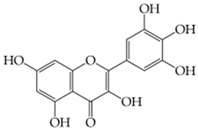 **Myricetin** (3,5,7-trihydroxy-2-(3,4,5-trihydroxyphenyl)-chromen-4-one)	Nanogel polymer: Chitosan Sonication technique Size: 236.2 ± 11.40 nm, PDI: 0.24 ± 0.02, zeta potential: 21.94 ± 0.51 mV, relative bioavailability 220.66%. The myricetin-loaded nanogels exhibited cell viability (Caco-2 cells) levels between 90% and 110% even after 24 h and 48 h of incubation, displayed a moderate and sustained release profile.	[144]
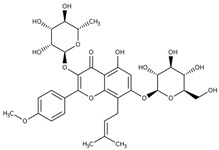 **Icariin** (5-hydroxy-2-(4-methoxyphenyl)-8-(3-methylbut-2-en-1-yl)-7-{[(2S,3R,4S,5S,6R)-3,4,5-trihydroxy-6-(hydroxymethyl)oxan-2-yl]oxy}-3-{[(2S,3R,4R,5R,6S)-3,4,5-trihydroxy-6-methyloxan-2-yl]oxy}-4H-chromen-4-one)	Reverse microemulsion technique Size = 73.80 ± 2.34 nm, PDI value < 0.15 indicating a narrow particle size distribution, zeta potential = −19.2 ± 1.14 mV, EE% = 2.03 ± 0.00%. In vitro, icariin nanogels released approximately 70% of icariin within 36 h, where the release followed zero-order kinetics for the first 8 h and a first-order release profile for the entire process. In animal behavioral studies, significantly reduced immobility time in the forced swim test (FST) and tail suspension test (TST) in mice, showing faster and better antidepressant-like effects compared to oral free-icariin administration. Nanogel formulation also restored testosterone, IL-6, and PGE2 levels in the plasma of CUMS (chronic unpredictable mild stress) rats more effectively than fluoxetine or icariin solution.	[145]
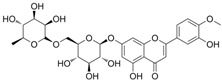 **Diosmin** (5-Hydroxy-2-(3-hydroxy-4-methoxyphenyl)-4-oxo-4H-chromen-7-yl 6-O-(6-deoxy-α-L-mannopyranosyl)-β-D-glucopyranoside)	Ionic gelation technique Size = 113.07 ± 12.62 nm, PDI = 0.266, zeta potential = 22.32 ± 0.56 mV, loading efficiency = 81.56 ± 2.65%, and loading capacity = 10.25 ± 1.43%. Exhibited swelling at pH 6.8 and 7.4, easily eroded at pH 1.2 and 4.5 (Fickian mechanism). Also, at pH 1.2 and pH 4.5, the equilibrium state was achieved with a 90% cumulative release in roughly 12 h. Mathematical models were used to fit in vitro release profiles to obtain an understanding of the drug release process.	[146]

## Data Availability

No new data were created or analyzed in this study. Data sharing is not applicable to this article.
